# Construction and practice of a multi-station Chinese medicine specialist nurse training system based on core competencies: a Delphi study

**DOI:** 10.3389/fmed.2026.1845170

**Published:** 2026-08-17

**Authors:** Hong Zhao, Yue Zhang, Yi Jia, Jing Wang, Zhaomei Wu, Chuanbing Huang

**Affiliations:** 1 Department of Nursing, The First Affiliated Hospital of Anhui University of Chinese Medicine, Hefei, Anhui, China; 2 School of Nursing, Anhui University of Chinese Medicine, Hefei, Anhui, China; 3 Department of Nursing, The Second Affiliated Hospital of Anhui University of Chinese Medicine, Hefei, Anhui, China

**Keywords:** Chinese medicine nurse specialists, Chinese medicine nurses, core competencies, Delphi, nurse specialists, training

## Abstract

**Background:**

With the increasing global recognition of traditional Chinese medicine (TCM), the standardized training of TCM specialist nurses remains challenging due to the lack of competency-based training frameworks that effectively integrate theoretical education with clinical practice. This study aimed to develop and evaluate a competency-based multi-station training system for TCM specialist nurses.

**Methods:**

A preliminary training framework was developed based on literature review, expert discussions, and the core competency framework of TCM specialist nurses. A three-round Delphi consultation involving 21 experts was conducted to refine the indicators, and the analytic hierarchy process was used to determine indicator weights. The effectiveness of the training system was evaluated among 40 trainees through pre- and post-training assessments of core competencies and satisfaction.

**Results:**

The Delphi consultation showed high expert authority and consensus, with authority coefficients ranging from 0.90 to 0.92. The final system comprised 6 primary indicators, 23 secondary indicators, and 122 tertiary indicators, with satisfactory consistency of indicator weights (CR < 0.10). After training, trainees demonstrated significant improvements in core competencies across multiple domains, including professional knowledge, clinical practice, management, innovation, teaching, communication, and ethical literacy (*P* < 0.05). High satisfaction with the training system was also reported.

**Conclusion:**

The competency-based multi-station training system provides a structured and practical approach for cultivating TCM specialist nurses. By integrating theoretical learning, clinical practice, and competency assessment, this model may contribute to the standardization of TCM nursing education and enhance nursing quality.

## 1 Introduction

With the evolution of global health concepts and the continuous transformation of medical and health systems, TCM has gained international recognition. Rooted in holistic principles, TCM provides comprehensive healthcare approaches (1, 2). Concurrently, by integrating holistic perspectives with individualized nursing approaches, TCM nursing has shown growing potential in chronic disease management, rehabilitation care, and geriatric health, thereby facilitating the delivery of comprehensive and personalized nursing services (3, 4). To further advance TCM nursing, its specialization and standardization have become central issues in the development of the healthcare system.

In recent years, the nursing discipline has been progressively advancing toward greater refinement and specialization. They have become a major direction of development in the international nursing field, meeting the growing health demands of the population and improving the quality of specialized nursing care (5, 6). As an important branch within the field of specialty nursing, TCM specialist nurses refer to nursing practitioners who have accumulated clinical experience, undergone systematic training to master TCM nursing techniques, and obtained certification through professional assessments (7). Their professional competence directly influences the clinical efficacy and disciplinary development of TCM nursing.

Although TCM specialist nurse training programs have gradually developed in China, a unified competency-based training framework has not yet been established. Existing programs mainly emphasize theoretical instruction and technical skills while lacking systematic integration of competency development with clinical practice (8). Therefore, developing a structured training model that effectively bridges theory and practice has become an urgent priority.

Competency-based training has been widely recognized as an effective approach for improving specialty nurse education by aligning training objectives with clinical practice requirements (9, 10). In addition, the multi-station training model proposed by Harden provides a structured framework for competency-based clinical education and assessment (11, 12). Although this model has been applied in specialty nursing education, its application in TCM specialist nurse training remains limited, and a standardized competency-based multi-station training system has yet to be established (13).

Given the distinctive characteristics of TCM nursing, specialist nurse education should effectively integrate traditional theoretical knowledge with competency-based clinical practice (14). However, existing training programs lack a standardized framework that systematically links competency requirements with curriculum design, clinical training, and assessment. Consequently, there remains a need for an evidence-based and structured training model that supports the standardized development of TCM specialist nurses.

Establishing a standardized training system has been proven to effectively improve training quality and accelerate the development of professional teams (15). Therefore, this study, grounded in the core competencies of TCM specialist nurses, adopts a university-hospital collaboration model to facilitate the sharing of foundational theories in TCM nursing education from universities. It also employs multi-station specialized clinical practice training and assessment, with an emphasis on mastering key nursing points for specific specialties and diseases, to enhance the clinical practice abilities of TCM specialist nurses and provide a reference for cultivating and managing core competencies among TCM specialist nurses in China.

## 2 Materials and methods

### 2.1 Construction of a training program for TCM specialist nurses

#### 2.1.1 Study design

The Delphi method has been widely utilized in fields such as business, military, education, and healthcare, and it leverages the expertise of experts to construct and enhance new research content (16). Its strength lies in harnessing the collective expertise of a panel to develop and refine research content. Through anonymous, iterative surveys, experts independently offer their perspectives and adjust them based on feedback, gradually moving toward consensus (17). This makes Delphi particularly suitable for research with limited empirical evidence or high uncertainty, especially in complex fields that require multidisciplinary expertise. At present, there is no established training program for TCM specialist nurses, and the construction of such a program requires the integration of TCM theory, clinical practice, and nursing education. Therefore, this study adopts the Delphi method to construct the training program for TCM specialist nurses.

#### 2.1.2 Establishment of the research group

The research group consisted of nine members, including one chief physician of TCM surgery, one chief superintendent nurse of TCM nursing, two deputy chief superintendent nurses specializing in TCM diabetes care, one professor of TCM nursing, one associate professor of TCM nursing, and three master’s students in nursing. In this study, the main responsibilities of the group members include: retrieving, screening, and synthesizing relevant literature; organizing expert panel discussions to develop the initial draft; drafting the consultation content, identifying the list of expert panel members, distributing and collecting questionnaires, conducting statistical analysis of the questionnaire results, and revising the indicators based on expert feedback; formulating the training program; and conducting preliminary implementation and effectiveness evaluation of the training system.

#### 2.1.3 Literature review

Following discussions within the research group, the following English search terms were used: “nurse/nurse specialist/nurse clinician/clinical nurse specialist,” “Medicine, Chinese Traditional/ Traditional Chinese Medicine/Chinese Medicine, Traditional,” and “competence/ability/capability/capacity/aptitude.” The search timeframe extended from the inception of each database to February 2025. The databases and websites searched included MEDLINE, Cochrane Library, Web of Science, PubMed, Embase, CINAHL, China National Knowledge Infrastructure (CNKI), Chinese Medical Journal Full-text Database, National Health Commission website, Wanfang Data, VIP Database, China Biomedical Literature Database (CBM), and Medlive, covering clinical practice guidelines, clinical decisions, and expert consensuses. Inclusion criteria were: (1) study subjects: TCM specialist nurses, with no restrictions on department, race, nationality, or gender; (2) study content: evaluation indicators related to the core competencies of TCM specialist nurses; (3) study context: studies conducted in settings such as hospitals, training bases, or schools using interviews, group discussions, or questionnaire administration; (4) study design: qualitative research employing methods such as the Delphi method, interview method, group discussion method, or expert evaluation method. Exclusion criteria were: (1) studies where subjects were not TCM specialist nurses, did not work in TCM medical institutions, or had not participated in TCM specialist nurses training; (2) literature that did not address core competency indicators for TCM specialist nurses; (3) duplicate publications; (4) literature not in Chinese or English; (5) studies for which full text was not available. A total of 2,335 articles were initially retrieved. After excluding duplicates, irrelevant studies, and those without full-text access, and considering the unique cultural and practical context of TCM nursing as well as the valuable international perspectives offered by English-language studies, 15 Chinese-language articles and one English-language article were ultimately included, resulting in a total of 16 articles. Among these, 13 studies employed the Delphi expert consultation method, and three used qualitative interviews. A systematic synthesis identified the core competencies of TCM specialist nurses, which include personal qualities, professional attributes, professional development capabilities, personal comprehensive abilities, organizational and management skills, professional knowledge and skills, and medical ethics and regulations.

#### 2.1.4 Group discussion on the research project

A research group was established, consisting of experts from universities and affiliated hospitals, including clinical medical and nursing specialists with senior titles, doctoral degrees, master’s degrees, and dual qualifications across multiple specialties. The members possess extensive experience in medical education and a profound and comprehensive understanding of the training of TCM specialist nurses. Building upon the integration of the core competencies of TCM specialist nurses, the research group expanded and deepened the training system with a focus on a multi-station, school-hospital collaborative training and assessment model. Additionally, the research group drew upon relevant, well-established nurse competency frameworks from both China and abroad, including the Chinese Registered Nurse Core Competency Framework, international advanced nursing practice models, international nurse practitioner core competency systems, and the current state of TCM nursing in China. Through group discussions, the team developed a preliminary draft of a multi-station TCM specialist nurses training system based on core competencies.

#### 2.1.5 Constructing consultation questionnaire

The expert consultation questionnaire consists of three parts:(1) A letter to the experts and a general information survey form, which explains the research background, purpose, and significance to the experts. (2) An expert consultation form for the multi-station TCM specialist nurse training system based on core competencies. Based on preliminary literature research and group discussions, the multi-station TCM specialist nurses training system based on core competencies was initially developed, comprising 6 first-level indicators (basic knowledge of TCM nursing, professional skills in TCM nursing, TCM clinical syndrome differentiation nursing, professional development ability, nursing management ability, and ethical and legal regulations), 21 second-level indicators, and 121 third-level indicators. The importance of the training indicators was evaluated using the Likert 5-level scale, and an expert comment section was provided for experts to add, delete, or modify items. (3) A quantitative scale for assessing the level of expert authority. See Supplementary Material 1 for specific training metrics.

#### 2.1.6 Selection of the experts

The Delphi panel size was determined purposively to ensure representation from TCM clinical nursing, TCM clinical medicine, nursing management, and nursing education while maintaining feasibility for iterative consultation. Twenty-one experts were recruited. To ensure the representativeness and reliability of the expert panel, this study employed purposive sampling to select 21 experts from provincial and municipal tertiary TCM hospitals and higher TCM institutions, specializing in TCM clinical practice, TCM clinical nursing, and TCM education. The selection criteria were as follows: (1) bachelor’s degree or above; (2) intermediate professional title or above; (3) at least 10 years of experience in TCM clinical nursing, TCM nursing education, TCM clinical practice, or nursing management; (4) active engagement in TCM research, strong motivation to participate, and willingness to cooperate with this study.

#### 2.1.7 Expert consultation process

Between May and June 2025, the researchers conducted consultations with experts who met the inclusion criteria via WeChat or email. Each round of collected questionnaires was reviewed promptly; any ambiguities or missing information were verified with the experts in a timely manner. After the first round of consultation, indicators were adopted or retained based on the following screening criteria: those with a mean importance score ≥3.5 and a coefficient of variation <0.25 (18). Combined with expert opinions, the items were further selected or revised after repeated deliberation by the research team, leading to the formation of the second-round consultation questionnaire. Following three rounds of expert consultation, opinions reached a consensus, and the consultation process was concluded.

### 2.2 Implementing a training program for specialized nurses in TCM

#### 2.2.1 Participants

A convenience sampling method was used to select 40 trainees who participated in the TCM specialist nurses training program in Anhui province in 2025 as the study subjects. (1) Inclusion criteria: meeting the registration requirements for TCM specialist nurses in Anhui province and successfully passing the selection examination. (2) Exclusion criteria: those who provided informed consent but did not agree to participate. (3) Elimination criteria: those who did not complete the questionnaire, provided logically inconsistent or extreme answers, completed the questionnaire in less than 2 min, or gave irrelevant responses.

#### 2.2.2 Research implementation

The constructed TCM specialist nurses training curriculum system was used as the theoretical course framework to provide theoretical training to the trainees. The course was scheduled for 5 weeks, with a total of 42 class hours. The course content was divided into theoretical training and practical operations, covering key areas such as basic TCM knowledge, TCM nursing professional skills, and TCM clinical syndrome differentiation nursing. The allocation of class hours was guided by the indicator weights. Based on the finalized multi-station TCM specialist nurse training system (covering 6 first-level indicators, 23 second-level indicators, and 122 third-level indicators), the research team mapped the indicator weights to the training content accordingly. Specifically, in terms of credit hour allocation, the ratio of theoretical to practical courses was set according to the weights of the first-level indicators–for example, basic TCM theory accounted for 40% of the total 42 credit hours, while TCM nursing skills accounted for 35%. Regarding teaching content design, each third-level indicator corresponded to a specific teaching module; for instance, “2.3 TCM nursing skills for Xiaoke (diabetes)” aligned with the practical training content at the diabetes TCM nursing station, which included tongue diagnosis, acupoint application, and dietary care. In terms of assessment methods, the evaluation content at each station was directly linked to the corresponding second-level indicators. For example, under “TCM clinical syndrome differentiation nursing,” the “clinical syndrome differentiation application of common appropriate TCM techniques” was assessed through a syndrome differentiation nursing station within the OSCE. Five stations were established based on the dominant TCM nursing specialties of the hospital, including TCM Specialized Nursing Clinic, diabetes TCM nursing, chronic obstructive pulmonary disease TCM nursing, cerebral infarction recovery stage and acute stage TCM nursing, and anal fistula TCM nursing. The content of each station was designed based on the third-level indicators under the second-level indicator “TCM nursing professional skills.” Each trainee rotated through each station for 1 week, completing the associated theoretical learning, skills training, and case discussions at the respective station. Each station was staffed with a TCM specialist nurse as an instructor, who delivered teaching in accordance with the *Station Teaching Manual*. A skills assessment was conducted at the conclusion of each station, and an objective structured clinical examination (OSCE) comprehensive assessment was administered after all rotations were completed.

#### 2.2.3 Evaluation methodology

(1) A self-designed satisfaction survey for clinical practice among TCM specialist nurses was used for evaluation. Satisfaction levels were rated using a 5-point Likert scale, with scores ranging from 1 (very dissatisfied) to 5 (very satisfied). The total score was 60, with higher scores indicating greater satisfaction.
(2) A self-designed core competency questionnaire for TCM specialist nurses was used to evaluate participants before and after the training. The assessment covered areas including TCM theoretical knowledge, TCM clinical nursing ability, nursing management ability, nursing innovation ability, nursing teaching ability, communication and coordination ability, professional development ability, and ethical and moral competence. A 5-point Likert scale was used to rate the level of core competency, with scores ranging from 1 (no understanding) to 5 (mastery). The total score was 515, with higher scores reflecting higher levels of core competency. The research team distributed and collected all questionnaires., achieving a 100% effective response rate.

#### 2.2.4 Data analysis

Excel and SPSS 26.0 were used for statistical analysis of the data. Descriptive analysis of expert information was conducted using frequencies, percentages, means, and standard deviations. Expert positivity was expressed as the questionnaire response rate. The expert authority coefficient was represented by the arithmetic mean of the judgment coefficient (Ca) and familiarity coefficient (Cs), with a Cr ≥ 0.7 generally considered acceptable reliability. Kendall’s coefficient of concordance ranges from 0 to 1; the closer the value is to 1, the higher the degree of coordination among expert opinions and the greater the consensus. The analytic hierarchy process was used to determine the weights of the indicators. The significance level was set at α = 0.05. A larger indicator weight indicates greater importance of the indicator within the overall system. Quantitative data conforming to a normal distribution were expressed as mean ± standard deviation (x ± s), and comparisons before and after training were performed using paired *t*-tests. Quantitative data not conforming to a normal distribution were expressed as median (M) and quartiles (P25, P75), with comparisons before and after training conducted using the Wilcoxon signed-rank test. The significance level was set at α = 0.05. *Post hoc* power analysis was performed using G*Power version 3.1.9.7. Achieved statistical power was calculated for paired-samples *t*-tests based on Cohen’s dz, with a two-sided significance level of 0.05 and a sample size of 40.

## 3 Results

### 3.1 Expert sociodemographic information

A total of 21 experts from tertiary first-class hospitals and universities in Anhui, Shanghai, Jiangsu, and other regions were included in this study. The average age of the experts was (47.33 ± 6.75) years, and 80.95% of them had a working experience of 20 years or more. There were 17 experts (80.95%) with deputy senior or above professional titles, and 4 (19.05%) with intermediate titles. Among them, 14 served as master’s student supervisors. The basic information of the experts is shown in Table 1.

**TABLE 1:** Basic information of experts (*n* = 21).

Groups	Classification	Number	Percent (%)
Age (years)	30∼39	2	9.52
	40∼49	11	52.38
	50∼59	7	33.33
	≥60	1	4.76
Highest education	Bachelor’s degree	14	66.67
	Master’s degree	5	23.81
	Doctor’s degree	2	9.52
Professional positions	Intermediate	4	19.05
	Associate senior	13	61.90
	Senior senior	4	19.05
Specialist areas	TCM clinical nursing	6	28.57
	TCM clinical medicine	2	9.52
	Nursing management	7	33.33
	Nursing education	6	28.57
Years of working	10∼19	4	19.05
	20∼29	8	38.10
	≥30	9	42.86
Serving as a graduate supervisor	No	7	33.33
	Yes	14	66.67

### 3.2 Enthusiasm and authority of experts

In the first and second rounds of consultation, 21 questionnaires were distributed each round, and 21 valid questionnaires were returned, with an effective response rate of 100.0% for both rounds. In the third round, 21 questionnaires were distributed, and 20 valid questionnaires were returned, resulting in an effective response rate of 95%. A total of 192 suggestions for modification were made in the first round, 96 in the second round, and 27 in the third round, summing to 315 suggestions, with an average of 15 suggestions per expert. This indicates that the experts demonstrated a high level of interest and active engagement in the construction of the multi-station TCM specialist nurse training system based on core competencies.

The expert authority level was represented by the arithmetic mean of the judgment basis and familiarity level. The assignment of the degree of influence (high/medium/low) for the expert judgment basis was as follows: theoretical analysis was assigned 0.3/0.2/0.1, practical experience was assigned 0.5/0.4/0.3, reference to domestic and international literature was assigned 0.1/0.1/0.1, and subjective judgment was assigned 0.1/0.1/0.1. The expert familiarity level was rated from very familiar to unfamiliar, with corresponding values of 1.0, 0.8, 0.5, 0.2, and 0, respectively. In the first round of this study, the judgment basis was 0.92, the familiarity level was 0.89, and the expert authority coefficient was 0.90. In the second round, the judgment basis was 0.94, the familiarity level was 0.92, and the expert authority coefficient was 0.93. In the third round, the judgment basis was 0.93, the familiarity level was 0.91, and the expert authority coefficient was 0.92. These results indicate that the experts participating in this study possessed a high level of authority and credibility.

### 3.3 Degree of coordination of expert opinions

The degree of coordination among expert opinions was expressed using the coefficient of variation (CV) and Kendall’s coefficient of concordance. The CV reflects the degree of variability in expert opinions on a specific indicator; a smaller CV indicates a higher level of coordination among experts regarding that indicator. Kendall’s coefficient of concordance ranges from 0 to 1, with a larger value indicating better coordination among experts. The CVs for all indicators across the three rounds of expert consultation ranged from 0 to 0.3, 0 to 0.23, and 0 to 0.2, respectively. After the first round of expert consultation, a total of 15 indicators had a CV > 0.20. Following adjustments to the training indicators based on expert opinions, two indicators had a CV > 0.20 in the second round, and in the third round, all indicators had a CV ≤ 0.20. Kendall’s coefficients of concordance for the three rounds ranged from 0.13 to 0.34, 0.12 to 0.15, and 0.11 to 0.21, respectively (*P* < 0.05), indicating that the experts reached consensus on the evaluation of the training indicators.

The Analytic Hierarchy Process (AHP) was applied to determine the weights of indicators based on the results of expert consultation. Judgment matrices were constructed, and consistency tests were performed to calculate the weights of indicators at different hierarchical levels. The derived weights represent the experts’ collective judgments regarding the relative importance of each indicator within the evaluation system; a higher weight indicates a greater relative contribution and importance of the corresponding indicator. The results are shown in Tables 2, 3.

**TABLE 2:** Degree of coordination of expert opinions across three rounds of consultation.

Items	Round 1			Round 2			Round 3		
	*W*	X^2^	*P*	*W*	X^2^	*P*	*W*	X^2^	*P*
Primary indicators	0.34	35.68	<0.001	0.15	16.00	<0.05	0.21	20.455	<0.001
Secondary indicators	0.16	66.52	<0.001	0.12	51.27	<0.001	0.11	49.445	<0.001
Tertiary indicators	0.13	328.25	<0.001	0.14	446.50	<0.001	0.11	279.487	<0.001

**TABLE 3:** Results of expert consultation on the multi-station TCM specialist nurses training system (third round).

Items	Approval rate (%)	Rating of importance (x̄ ± s)	Coefficient of variation	Weighting target
A. Basic knowledge of TCM nursing	100.00	5.00 ± 0.00	0.000	0.304
A1. Basic theory of TCM	85.00	4.85 ± 0.37	0.075	0.057
A2. Basic theory of TCM nursing	95.00	4.95 ± 0.22	0.045	0.110
B. Professional skills in TCM nursing	100.00	5.00 ± 0.00	0.000	0.304
B1. General clinical TCM appropriate techniques	85.00	4.85 ± 0.37	0.075	0.057
B2. Risk prevention and management in TCM nursing skills	85.00	4.80 ± 0.52	0.109	0.040
B3. TCM nursing skills for Xiaoke	80.00	4.80 ± 0.41	0.085	0.040
B4. TCM nursing for lung distention	75.00	4.75 ± 0.44	0.093	0.029
B5. TCM nursing skills for the acute and recovery stages of stroke	75.00	4.75 ± 0.44	0.093	0.029
B6. TCM nursing skills for anal fistula	65.00	4.60 ± 0.60	0.130	0.012
B7. TCM nursing skills for preventive treatment	85.00	4.85 ± 0.37	0.075	0.057
B8. TCM home wellness care skills	75.00	4.75 ± 0.44	0.093	0.029
C. TCM clinical syndrome differentiation nursing	95.00	4.95 ± 0.22	0.045	0.202
C1. Clinical application of common TCM appropriate techniques based on syndrome differentiation	95.00	4.95 ± 0.22	0.045	0.110
C2. Syndrome differentiation-based care for common clinical conditions	95.00	4.95 ± 0.22	0.045	0.110
D. Professional development competence	85.00	4.80 ± 0.52	0.109	0.089
D1. Nursing research competency	75.00	4.60 ± 0.82	0.178	0.012
D2. Teaching ability	65.00	4.60 ± 0.60	0.130	0.012
D3. Innovation capability	75.00	4.70 ± 0.57	0.121	0.022
D4. Critical thinking	65.00	4.65 ± 0.49	0.105	0.016
D5. Self-directed learning ability	75.00	4.75 ± 0.44	0.093	0.028
E. Nursing management competence	65.00	4.60 ± 0.60	0.130	0.034
E1. TCM Nursing quality management	70.00	4.55 ± 0.83	0.182	0.009
E2. Organizational and leadership abilities	60.00	4.40 ± 0.88	0.200	0.007
E3. Communication, collaboration, and coordination abilities	75.00	4.65 ± 0.67	0.144	0.020
F. Ethical and moral regulations	80.00	4.75 ± 0.55	0.116	0.067
F1. Humanistic care	85.00	4.85 ± 0.37	0.075	0.056
F2. Ethics and morality	85.00	4.85 ± 0.37	0.075	0.058
F3. Medical regulations (supplement)	90.00	4.90 ± 0.31	0.063	0.079

Approval rate (%) indicates the proportion of experts who rate this indicator 5 points, that is “very important”.

### 3.4 Experts’ opinions on the training course

In the first round of expert consultation, a total of 192 comments and suggestions were received. After thorough discussion by the coordination group, 150 were adopted, 20 were partially adopted, and 22 were not adopted. In the second round, 96 comments and suggestions were received, of which 75 were adopted, and 21 were not adopted. In the third round, 27 comments and suggestions were received, with 17 adopted and 10 not adopted. The written suggestions mainly included modifications to the expression of TCM terminology, additions of training indicators, deletion of duplicate or low-relevance indicators, merging of similar indicators, and expansion of indicator scope. Suggestions for textual modifications included changing Western medicine disease names to TCM disease names–for example, changing “diabetes” to “Xiaoke,” and “chronic obstructive pulmonary disease” to “lung distension” (adopted); and changing the third-level indicator “syndrome differentiation” to “eight-principle syndrome differentiation.” Suggestions for adding training indicators included adding the second-level indicator “communication and coordination skills” under the first-level indicator “nursing management ability” (adopted); and adding the second-level indicator “medical regulations” under the first-level indicator “ethical and legal regulations” (adopted). Suggestions for deleting indicators included recommending the removal of “acupuncture technique” as it falls outside the scope of nursing techniques (adopted). Suggestions for merging indicators included combining “TCM self-care” and “sub-health regulation” into “sub-health TCM self-care”; and merging “fire dragon moxibustion,” “umbilical moxibustion,” and “governor vessel moxibustion” into “moxibustion therapy.” Suggestions for expanding the scope of indicators included adding “TCM exercises” to “commonly used appropriate TCM techniques.” Additionally, based on expert recommendations, indicators with inconsistent terminology, improper sequencing, unclear prioritization, excessive granularity, or a coefficient of variation (CV) greater than 0.2 were discussed and revised by the group. Ultimately, a training indicator system for TCM specialist nurses was formed, comprising 6 first-level indicators, 23 second-level indicators, and 122 third-level indicators.

### 3.5 Evaluation of training effects before and after

#### 3.5.1 General information of the study subjects

Forty trainees participated in the study, including one male (2.5%) and 39 females (97.5%). Their mean age was 33.50 ± 3.52 years (range: 27–42 years). In terms of professional titles, there were 11 junior nurses (27.50%), 27 supervisor nurses (67.50%), and 2 associate chief nurses (5.00%). Regarding positions, there was 1 head nurse (2.50%), 1 deputy head nurse (2.50%), and 38 staff nurses (95.00%). In terms of educational background, 3 participants held an associate degree (7.50%), 35 held a bachelor’s degree (87.50%), and 2 held a master’s degree (3.33%).

#### 3.5.2 Pre-post comparison of core competency scores among TCM specialist nurses

As shown in Table 4, all competency domains demonstrated higher scores after completion of the competency-based multi-station training program than before training. After Bonferroni correction, almost between-time comparisons remained statistically significant (adjusted *P* < 0.00625). Overall, the intervention improved all assessed competency domains.

**TABLE 4:** Pre-post comparison of core competency scores among TCM specialist nurses.

Competency domain	Pre-training (40)	Post-training (40)	95% CI	Statistic	*P*	Cohen’s dz	Estimated power
TCM theoretical knowledge	2.23 ± 0.53^a^	3.55 ± 0.62^a^	(1.11, 1.54)	−12.46^c^	<0.001	1.97	>0.999
Clinical nursing competence in TCM	2.13 ± 0.59^a^	3.55 ± 0.62^a^	(1.19, 1.68)	−11.77^c^	<0.001	1.86	>0.999
Nursing management competence	2.33 ± 0.66^a^	3.50 ± 0.73^a^	(0.92, 1.41)	−9.67^c^	<0.001	1.53	>0.999
Nursing innovation ability	1.93 ± 0.68^a^	3.33 ± 0.73^a^	(1.12, 1.68)	−9.98^c^	<0.001	1.58	>0.999
Nursing teaching competency	2.48 ± 0.86^a^	3.54 ± 0.79^a^	(0.79, 1.33)	−7.96^c^	<0.001	1.26	0.999
Professional development competence	3.35 ± 1.07^a^	4.00 ± 0.79^a^	(0.31, 1.00)	−3.89^c^	<0.001	0.62	0.964
Communication and coordination skills	3.50 (3.00,4.00)^b^	4.00 (3.42,4.92)^b^	NA	−2.55^d^	0.011	0.59	0.84
Ethical and moral competence	4.20 (3.20,4.75)^b^	4.80 (4.00,5.00)^b^	NA	−2.88^d^	0.004	0.58	0.91

^a^Indicates quantitative data conforming to a normal distribution, expressed as mean ± standard deviation (x̄ ± s);

^b^Indicates quantitative data not conforming to a normal distribution, expressed as median (M) and quartiles (P25, P75);

^c^Indicates the *t*-value obtained from the paired sample *t*-test;

^d^Indicates the *Z*-value obtained from the Wilcoxon signed-rank test.

Cohen’s dz was calculated as the mean paired difference divided by the standard deviation of paired differences. Large effect sizes were observed for theoretical knowledge and TCM clinical nursing competence, whereas moderate effects were found for professional development, communication, and ethical competence.

Power values were estimated based on Cohen’s dz. *Post hoc* power exceeded 0.90 for all paired *t*-test outcomes, while the Wilcoxon-based outcomes demonstrated acceptable statistical power (>0.80), indicating that the current sample size was sufficient to detect the observed intervention effects.

#### 3.5.3 Scores of clinical practice satisfaction among TCM specialist nurses after training

Following completion of the competency-based multi-station training program, trainees’ satisfaction with clinical practice was evaluated. Overall, participants reported high satisfaction with the training experience. As shown in Table 5, the median satisfaction scores for the training base, clinical instructors, and other aspects were 4.50 (4.25, 4.75), 4.71 (4.57, 4.86), and 5.00 (4.00, 5.00), respectively, indicating that the training system was well accepted by TCM specialist nurses in terms of clinical practice organization, instructor support, and overall training implementation.

**TABLE 5:** Satisfaction scores of TCM specialist nurses with clinical practice after training (unit: points).

Items	Minimum	Maximum	M (P25, P75)
Satisfaction with the training base	3.50	5.00	4.50 (4.25, 4.75)
Satisfaction with clinical instructors	4.00	5.00	4.71 (4.57, 4.86)
Satisfaction with other aspects	4.00	5.00	5.00 (4.00, 5.00)

## 4 Discussion

Through long-term practical experience, TCM nursing has developed a system of non-pharmacological therapies that can be applied not only to the clinical treatment of diseases, but also as a health maintenance approach to be promoted among community residents (19, 20). This study is grounded in the current demand for nursing talent with distinctive characteristics of TCM and the current state of the industry. Through a systematic review of academic materials from both China and abroad, a comprehensive integration of the core competencies of TCM specialist nurses was achieved. In constructing the theoretical framework, established models such as the Chinese Registered Nurse Core Competency Standards, the Strong Advanced Practice Nursing Model, and the International NP Competency Framework were comprehensively referenced, leading to the innovative development of a multi-station TCM specialist nurses training program based on core competencies. The expert consultation panel covered the two major fields of tertiary hospitals and higher education institutions, with selection criteria emphasizing both clinical and teaching expertise (81% of experts had over 20 years of experience in clinical or teaching practice). In applying the Delphi method, the study focused on three core elements: the representativeness of the expert structure, the timeliness of questionnaire feedback, and the authority of academic judgment (21). The expert positivity rates across the three rounds were 100%, 100%, and 95%, respectively; the authority coefficients were 0.9, 0.93, and 0.93; and the familiarity with the indicator system was 0.89, 0.92, and 0.92, respectively. The coefficients of variation for the indicator system ranged from 0 to 0.3, 0 to 0.23, and 0 to 0.2 across the three rounds, indicating a high level of authority and representativeness among the expert opinions. This training system, built upon the core competencies of TCM specialist nurses, adopts a multi-station, integrated training approach involving both schools and hospitals. It emphasizes the cultivation of clinical practice skills among TCM specialist nurses alongside theoretical knowledge in TCM nursing. Through systematic rotation training and practical application at multiple stations, trainees improved their professional knowledge and clinical skills (22). Research by Cao Haiying et al. (23) suggests that scientifically sound curriculum design, diversified training methods, and robust evaluation and feedback mechanisms can significantly improve the effectiveness of TCM specialist nurses training. Therefore, on the basis of theoretical training, this study adopts multi-station clinical practice training in specialized departments, which can better help TCM specialist nurses deepen their understanding of knowledge and master the key points of specialized disease nursing.

Indicator weights are crucial in the construction of an indicator system. The results show that the CR value for first-level indicators is 0.053, and for second-level indicators, it is 0.051, both of which are less than 0.1. Generally, a smaller CR value indicates better consistency of the judgment matrix, demonstrating that the weight assignment for each indicator is reasonable (24). Among the first-level indicators, the highest weights are assigned to Basic Knowledge of TCM Nursing and Professional Skills in TCM Nursing (0.304), indicating that experts believe specialist nurses in TCM should focus training on basic TCM knowledge and TCM nursing skills, which aligns with previous studies (23). This also corresponds with our training approach, which emphasizes deepening the clinical nursing skills of TCM specialist nurses based on a solid foundation of theoretical knowledge in TCM nursing, achieving the fundamental goal of applying what has been learned. Multiple domestic and international studies have shown (25, 26) that a solid knowledge base is a necessary condition for developing nurses’ core competencies. Similarly, the core competencies of TCM specialist nurses are highly dependent on a strong foundation of basic theoretical knowledge. Among the first-level indicators, nursing management ability has a relatively low weight (0.034), which differs from the training of advanced practice nurses abroad (27). The reason may be that experts believe TCM specialist nurses should focus more on basic knowledge and skills to serve clinical patients effectively. Additionally, the training of TCM specialist nurses in China started relatively late, so this aspect has not yet been fully emphasized. Among the second-level indicators, the Clinical Application of Common TCM Appropriate Techniques Based on Syndrome Differentiation and Syndrome Differentiation-Based Care for Common Clinical Conditions (0.110) have higher weights, indicating that experts generally believe mastering TCM appropriate techniques and syndrome differentiation-based care are the priorities of training. Therefore, the training duration for this part could be increased during the cultivation process, and systematic, targeted practical teaching should be used to strengthen this core competency, better meeting the demands of clinical nursing.

This study shows that the scores for various core competency indicators of TCM specialist nurses after training were higher than those before training, and the differences were statistically significant, which is similar to the findings of related research (28). This may be attributed to the fact that the preliminary phase of this study was based on the integration of core competencies of TCM specialist nurses, and expert group discussions were conducted based on a mature core competency system. Additionally, a multi-station training system for TCM specialist nurses was specifically constructed through three rounds of expert consultations, utilizing a combined college-hospital training model with multi-station assessment. Unlike previous studies (29), firstly, this TCM specialist nurses training system adopts a college-hospital collaboration model, achieving the sharing of theoretical resources in TCM nursing education from universities, integrating TCM culture, contributing to the inheritance of TCM classics, and promoting new advances in TCM nursing and technological innovation research. Secondly, it closely integrates with clinical practice in hospitals, aiming to enhance the clinical application abilities of TCM specialist nurses. Finally, it highlights the characteristics of multi-station specialized disease training, establishing five advantageous training stations: nursing care for consumptive thirst (type 2 diabetes mellitus), lung distension (chronic obstructive pulmonary disease), stroke (cerebral infarction recovery phase, cerebral infarction acute phase), anal fistula disease (anal fistula), and a specialized TCM nursing clinic. This can stimulate interest and motivation in practical learning, cultivate clinical TCM nursing thinking, and comprehensively improve professional competence. The multi-station training system is an objective, orderly, and structured framework proposed by foreign medical experts and has been widely applied in nursing education in developed countries. It uses simulated clinical scenarios to train and assess the clinical skills of students and nurses (30, 31). In recent years, some medical colleges and hospitals in China have also applied the multi-station nurse training and assessment system, achieving positive results. This system strengthens the application of TCM nursing knowledge and completes practical skills training based on the characteristics of specific departmental diseases (32). The multi-station training model encompasses multiple aspects, allowing for comprehensive and systematic learning of TCM knowledge and skills. Through integrated training, it stimulates nurses’ learning interest, cultivates clinical TCM nursing thinking, enables them to better understand TCM theory, and apply it in practice (33).

The major distinction of this training system from previous Chinese medicine specialist nurse programs is its competency-oriented, multi-station design, which integrates university-based theoretical resources with hospital clinical practice. Unlike traditional models that rely mainly on lectures and general skill training, this system incorporates disease-specific stations (e.g., Xiaoke, lung distension, stroke, and anal fistula) to provide case-based and practice-oriented learning, effectively bridging the gap between theory and clinical application. It enhances nurses’ competencies in TCM knowledge, syndrome differentiation nursing, and professional practice, while offering a standardized and replicable framework for TCM specialist nurses training. Furthermore, this system addresses the limitations of previous training models by establishing an objective and structured competency-based curriculum that can better support improvements in clinical nursing quality. Moreover, the development and implementation of this training system align with the World Health Organization’s global strategy on traditional and complementary medicine (2014–2023) and related recommendations, which encourage the integration of traditional medicine into national healthcare systems, particularly in settings where accessible and affordable healthcare is needed (34). TCM nursing techniques, including acupoint application, moxibustion, and dietary care, are characterized by low cost and limited reliance on advanced equipment, making them applicable to primary care and community health services. By systematically developing TCM specialist nurses with enhanced clinical competencies and evidence-based decision-making skills, this training system provides a feasible approach to improving the quality of TCM nursing services, especially in resource-limited areas. Although the current system was developed within the Chinese TCM healthcare context, its competency-based design, structured curriculum development, Delphi consensus methodology, and OSCE-based assessment framework are not limited to TCM. With appropriate localization of disease content, educational resources, and clinical competencies, this framework could be adapted for specialist nursing education in Southeast Asia, Africa, and other regions where traditional medicine is integrated into primary healthcare services.

Furthermore, it offers a potential reference for integrating traditional medicine into contemporary healthcare systems. The findings highlight the translational value of this competency-based training framework in advancing both individual nursing practice and broader healthcare integration.

Because this preliminary study adopted a single-group pre-post design without a concurrent control group, the observed improvements cannot be solely attributed to the intervention. Alternative explanations, including maturation effects, repeated testing, increased familiarity with assessment procedures, and the Hawthorne effect, may also have contributed to the observed changes. Therefore, the present findings should be interpreted as preliminary evidence of feasibility rather than definitive evidence of effectiveness.

## 5 Limitation

Despite the promising progress achieved in this study on the training of TCM specialist nurses, some limitations still need to be acknowledged. First, the preliminary implementation adopted a single-group pre-post design without a concurrent control group; therefore, causal attribution is limited. Second, participants were recruited from a single provincial training cohort using convenience sampling, which may introduce selection bias and limit generalizability. Third, the evaluation was conducted immediately after training without long-term follow-up; consequently, the retention and transfer of competencies to clinical practice remain unknown. Fourth, the primary competency outcome was based on self-reported assessment and may be influenced by response bias. Additionally, the two primary questionnaires assessing satisfaction and competencies used in this study was self-developed for the specific training context and lacked systematic pilot testing and psychometric validation, including Cronbach’s α and factor analysis. Future studies should formally validate these instruments by evaluating internal consistency, construct validity, and test-retest reliability before broader application. Finally, although the framework was developed through multidisciplinary expert consultation, further validation across diverse geographical and institutional settings is required.

## 6 Conclusion

This study developed a competency-based multi-station training system for TCM specialist nurses through expert consensus and practical evaluation. The proposed framework integrates theoretical education, clinical practice, skills training, and competency assessment, providing a structured approach for specialist nurse development. Further multicenter studies with larger samples and longer follow-up periods are warranted to validate its effectiveness and applicability in different healthcare settings.

## Data Availability

The original contributions presented in this study are included in this article/Supplementary material, further inquiries can be directed to the corresponding author.
