# Supplementary material for: Construction and practice of a multi-station Chinese medicine specialist nurse training system based on core competencies: a Delphi study
Source: Front Med (Lausanne). 2026 Aug 17;13:1845170. doi: 10.3389/fmed.2026.1845170 (PMC13531516; doi:10.3389/fmed.2026.1845170)
Supplement: Supplementary file 1 [file Table_1.docx]

**Supplementary Content**

**Supplementary Material 1:**

**Construction of Training Curriculum for Traditional Chinese Medicine (TCM) Specialist Nurse Training Base**

Delphi Expert Consultation Questionnaire (Round 3)

Dear Experts,

Greetings!We sincerely appreciate your participation in completing the third-round Delphi expert consultation questionnaire for this study. Your valuable feedback in the first and second rounds provided essential guidance for this research.

After collecting and reviewing all experts’ suggestions, we carefully discussed and revised the training curriculum indicators. The modifications are as follows: the number of first-level indicators remains unchanged; the number of second-level indicators has increased from 22 to 23; and the number of third-level indicators has been reduced from 156 to 122.We hereby present the revised curriculum framework of the TCM Specialist Nurse Training Base and invite you to participate in this third-round expert consultation. The purpose of this round is to evaluate the importance and necessity of the revised indicators.

This questionnaire consists of three parts:

1. General information of experts

2. Expert consultation form on curriculum content for TCM specialist nurse training base

3. Basis of judgment and familiarity with the subject

To ensure the smooth progress of this study, we kindly ask you to complete all items carefully upon receipt. Your personal information and responses will be used solely for academic research and will be kept strictly confidential.

Thank you again for your support and contribution.We wish you a happy and prosperous New Year!

The First Affiliated Hospital of Anhui University of Chinese Medicine

December 2024

Contact Person: Bai Ling

Tel: 0551-62838593

Project Leader: Zhao Hong

**Part I. General Information of Experts**

Dear Experts,

This survey is intended solely for analyzing the overall characteristics of participating experts. All information will be strictly confidential. Please complete the form or tick “√” in the appropriate boxes.

| Name |  | Age (years) |  | Highest Degree | 🞎Bachelor  🞎Master 🞎PhD |
| --- | --- | --- | --- | --- | --- |
| Professional Title |  | Email |  | Telephone |  |
| Supervisor Status | 🞎Yes（🞎Master supervisor 🞎Doctoral supervisor）  🞎No | | | | |
| Field of Work (multiple choices allowed) | | | | 🞎Clinical Nursing 🞎Clinical Medicine  🞎Sports and Health Sciences  🞎Psychology  🞎Statistics 🞎Community Nursing  🞎Clinical Management 🞎Clinical Education  🞎Other: _________ | |
| Years of Professional Experience: | | | | 🞎10～19years  🞎20～29years  🞎≥30 years | |
| Have you ever led or participated in research on specialist nurse training? | | | | 🞎Yes 🞎No | |
| Have you ever led or participated in training or lectures on TCM specialist nursing? | | | | 🞎Yes 🞎No | |

**Part II. Delphi Consultation Form for Curriculum Content of TCM Specialist Nurse Training Base**

Instructions:

1. This questionnaire is based on the revised second-round Delphi results, including:

6 first-level indicators;23 second-level indicators;122 third-level indicators

2. Please rate the importance of each item using a 5-point Likert scale:

3. You may suggest deletion, modification, or addition of items in the comment section, or indicate “None”.

**Table 1. Expert Judgment Basis and Familiarity**

| Basis of judgment for importance evaluation | Basis | | | Please indicate the influence level | | | | | |
| --- | --- | --- | --- | --- | --- | --- | --- | --- | --- |
|  |  |  |  | High | | Medium | | Low | |
|  | 1.Practical experience | | | 🞎 | | 🞎 | | 🞎 | |
|  | 2.Theoretical analysis | | | 🞎 | | 🞎 | | 🞎 | |
|  | 3.Domestic and international literature | | | 🞎 | | 🞎 | | 🞎 | |
|  | 4.Subjective judgmen | | | 🞎 | | 🞎 | | 🞎 | |
| Familiarity with the content | | Level | | | | | | | |
|  |  | Very familiar | Relatively familiar | | Moderately familiar | | Slightly familiar | | Not familiar |
| Basic theory | | 🞎 | 🞎 | | 🞎 | | 🞎 | | 🞎 |
| Basic knowledge | | 🞎 | 🞎 | | 🞎 | | 🞎 | | 🞎 |
| Basic skills | | 🞎 | 🞎 | | 🞎 | | 🞎 | | 🞎 |

**Table 2. Evaluation of First-Level Indicators**

| First-Level Indicators | Please rate importance (1–5) | | | | | expert opinion |
| --- | --- | --- | --- | --- | --- | --- |
|  | 1 | 2 | 3 | 4 | 5 |  |
| 1.Basic knowledge of TCM nursing | 🞎 | 🞎 | 🞎 | 🞎 | 🞎 |  |
| 2.Professional skills of TCM nursing | 🞎 | 🞎 | 🞎 | 🞎 | 🞎 |  |
| 3.TCM clinical syndrome differentiation nursing | 🞎 | 🞎 | 🞎 | 🞎 | 🞎 |  |
| 4.Professional development ability | 🞎 | 🞎 | 🞎 | 🞎 | 🞎 |  |
| 5.Nursing management ability | 🞎 | 🞎 | 🞎 | 🞎 | 🞎 |  |
| 6.Ethics and legal regulations | 🞎 | 🞎 | 🞎 | 🞎 | 🞎 |  |
| Comments： | | | | | | |

**Table 3. Evaluation of Second-Level Indicators**

| First-Level Indicators | Second-Level Indicators | Please rate importance (1–5) | | | | | expert opinion |
| --- | --- | --- | --- | --- | --- | --- | --- |
|  |  | 1 | 2 | 3 | 4 | 5 |  |
| 1.Basic knowledge of TCM nursing | 1.1 Basic theories of TCM | 🞎 | 🞎 | 🞎 | 🞎 | 🞎 |  |
|  | 1.2 Fundamental theories of TCM nursing | 🞎 | 🞎 | 🞎 | 🞎 | 🞎 |  |
|  | Comments： | | | | | | |
| 2.Professional skills of TCM nursing | 2.1Common TCM appropriate technologies | 🞎 | 🞎 | 🞎 | 🞎 | 🞎 |  |
|  | 2.2 Risk prevention and management of TCM nursing techniques | 🞎 | 🞎 | 🞎 | 🞎 | 🞎 |  |
|  | 2.3TCM nursing for diabetes mellitus (Xiaoke disease) | 🞎 | 🞎 | 🞎 | 🞎 | 🞎 |  |
|  | 2.4 TCM nursing for COPD (Feizhang syndrome) | 🞎 | 🞎 | 🞎 | 🞎 | 🞎 |  |
|  | 2.5 TCM nursing for stroke (acute and recovery stages) | 🞎 | 🞎 | 🞎 | 🞎 | 🞎 |  |
|  | 2.6 TCM nursing for anal fistula | 🞎 | 🞎 | 🞎 | 🞎 | 🞎 |  |
|  | 2.7Preventive treatment of disease (Zhi Wei Bing) nursing techniques | 🞎 | 🞎 | 🞎 | 🞎 | 🞎 |  |
|  | 2.8Home-based TCM health maintenance nursing skills | 🞎 | 🞎 | 🞎 | 🞎 | 🞎 |  |
|  | Comments： | | | | | | |
| 3.TCM clinical syndrome differentiation nursing | 3.1 Clinical application of TCM appropriate techniques | 🞎 | 🞎 | 🞎 | 🞎 | 🞎 |  |
|  | 3.2 Syndrome differentiation-based nursing of common diseases | 🞎 | 🞎 | 🞎 | 🞎 | 🞎 |  |
|  | Comments： | | | | | | |
| 4.Professional development ability | 4.1 Nursing research ability | 🞎 | 🞎 | 🞎 | 🞎 | 🞎 |  |
|  | 4.2 Teaching ability | 🞎 | 🞎 | 🞎 | 🞎 | 🞎 |  |
|  | 4.3 Innovation ability | 🞎 | 🞎 | 🞎 | 🞎 | 🞎 |  |
|  | 4.4 Critical thinking ability | 🞎 | 🞎 | 🞎 | 🞎 | 🞎 |  |
|  | 4.5 Self-directed learning ability | 🞎 | 🞎 | 🞎 | 🞎 | 🞎 |  |
|  | Comments： | | | | | | |
| 5.Nursing management ability | 5.1 TCM nursing quality management | 🞎 | 🞎 | 🞎 | 🞎 | 🞎 |  |
|  | 5.2 Organizational and leadership ability | 🞎 | 🞎 | 🞎 | 🞎 | 🞎 |  |
|  | 5.3Communication, coordination, and teamwork ability |  |  |  |  |  |  |
|  | Comments： | | | | | | |
| 6.Ethics and legal regulations | 6.1 Humanistic care | 🞎 | 🞎 | 🞎 | 🞎 | 🞎 |  |
|  | 6.2Ethical principles | 🞎 | 🞎 | 🞎 | 🞎 | 🞎 |  |
|  | 6.3 Medical laws and regulations | | | | | | |
|  | Comments： | | | | | | |

**Table 4. Evaluation of Third-Level Indicators**

| First-Level Indicators | Second-Level Indicators | **Third-Level Indicators** | Please rate importance (1–5) | | | | | expert opinion |
| --- | --- | --- | --- | --- | --- | --- | --- | --- |
|  |  |  | 1 | 2 | 3 | 4 | 5 |  |
| 1.Basic knowledge of TCM nursing | 1.1 Basic theories of TCM | 1.1.1 Brief history of TCM development and theoretical system | 🞎 | 🞎 | 🞎 | 🞎 | 🞎 |  |
|  |  | 1.1.2 Yin-Yang and Five Elements theory | 🞎 | 🞎 | 🞎 | 🞎 | 🞎 |  |
|  |  | 1.1.3Zang-Xiang theory (organ manifestation theory) | 🞎 | 🞎 | 🞎 | 🞎 | 🞎 |  |
|  |  | 1.1.4 Meridians and acupoints | 🞎 | 🞎 | 🞎 | 🞎 | 🞎 |  |
|  |  | 1.1.5 Qi, Blood, Body Fluids theory | 🞎 | 🞎 | 🞎 | 🞎 | 🞎 |  |
|  |  | 1.1.6Fundamentals of prescriptions and herbal medicine | 🞎 | 🞎 | 🞎 | 🞎 | 🞎 |  |
|  |  | 1.1.7 Etiology and basic pathogenesis | 🞎 | 🞎 | 🞎 | 🞎 | 🞎 |  |
|  |  | 1.1.8Eight principles of syndrome differentiation | 🞎 | 🞎 | 🞎 | 🞎 | 🞎 |  |
|  |  | 1.1.9Four diagnostic methods (inspection, auscultation & olfaction, inquiry, palpation) | 🞎 | 🞎 | 🞎 | 🞎 | 🞎 |  |
|  |  | 1.1.10 Introduction to classical TCM texts |  |  |  |  |  |  |
|  |  | Comments： | | | | | | |
|  |  | 1.2.1 Principles and characteristics of TCM nursing | 🞎 | 🞎 | 🞎 | 🞎 | 🞎 |  |
|  |  | 1.2.2 Daily life nursing in TCM | 🞎 | 🞎 | 🞎 | 🞎 | 🞎 |  |
|  |  | 1.2.3 Dietary nursing in TCM | 🞎 | 🞎 | 🞎 | 🞎 | 🞎 |  |
|  |  | 1.2.4 Medication nursing in TCM | 🞎 | 🞎 | 🞎 | 🞎 | 🞎 |  |
|  |  | 1.2.5 Emotional nursing in TCM | 🞎 | 🞎 | 🞎 | 🞎 | 🞎 |  |
|  |  | 1.2.6Constitution-based nursing and condition observation | 🞎 | 🞎 | 🞎 | 🞎 | 🞎 |  |
|  |  | 1.2.7Health education in TCM |  |  |  |  |  |  |
|  |  | Comments： | | | | | | |
| 2.Professional skills in traditional Chinese medicine nursing | 2.1 Commonly used appropriate techniques of traditional Chinese medicine | 2.1.1 Auricular acupressure | 🞎 | 🞎 | 🞎 | 🞎 | 🞎 |  |
|  |  | 2.1.2 Scraping therapy (Gua Sha) | 🞎 | 🞎 | 🞎 | 🞎 | 🞎 |  |
|  |  | 2.1.3 Bloodletting cupping therapy | 🞎 | 🞎 | 🞎 | 🞎 | 🞎 |  |
|  |  | 2.1.4Bloodletting therapy (e.g., ear apex bleeding) | 🞎 | 🞎 | 🞎 | 🞎 | 🞎 |  |
|  |  | 2.1.5 Herbal dressing change | 🞎 | 🞎 | 🞎 | 🞎 | 🞎 |  |
|  |  | 2.1.6Acupoint application | 🞎 | 🞎 | 🞎 | 🞎 | 🞎 |  |
|  |  | 2.1.7Acupressure massage | 🞎 | 🞎 | 🞎 | 🞎 | 🞎 |  |
|  |  | 2.1.8 Moxibustion |  |  |  |  |  |  |
|  |  | 2.1.9 TCM exercise therapy |  |  |  |  |  |  |
|  |  | Comments： | | | | | | |
|  | 2.2Risk prevention and management | 2.2.1 Prevention and management of adverse events (burns, infection, syncope, etc.) | 🞎 | 🞎 | 🞎 | 🞎 | 🞎 |  |
|  |  | 2.2.2 Infection control in TCM nursing operations | 🞎 | 🞎 | 🞎 | 🞎 | 🞎 |  |
|  |  | Comments： | | | | | | |
|  | 2.3 Traditional Chinese Medicine Nursing for Diabetes | 2.3.1 Moxibustion for the prevention and intervention of diabetic foot | 🞎 | 🞎 | 🞎 | 🞎 | 🞎 |  |
|  |  | 2.3.2 Prevention and intervention of diabetic ocular complications through traditional Chinese medicine ocular nebulization | 🞎 | 🞎 | 🞎 | 🞎 | 🞎 |  |
|  |  | 2.3.3 The technical application of traditional Chinese medicine soaking and washing for diabetic foot of different Wagner grades | 🞎 | 🞎 | 🞎 | 🞎 | 🞎 |  |
|  |  | 2.3.4 The Application of Traditional Chinese Dietary Therapy formulas in Diabetic diets | 🞎 | 🞎 | 🞎 | 🞎 | 🞎 |  |
|  |  | 2.3.5 The principle of the operation essentials of the Baduanjin Gong Method in improving the symptoms of diabetes | 🞎 | 🞎 | 🞎 | 🞎 | 🞎 |  |
|  |  | Comments： | | | | | | |
|  | 2.4 Traditional Chinese Medicine Nursing for chronic obstructive pulmonary disease | 2.4.1 The application of umbilical moxibustion therapy in patients with lung and spleen Qi deficiency and diarrhea in the respiratory department | 🞎 | 🞎 | 🞎 | 🞎 | 🞎 |  |
|  |  | 2.4.2 The Application of the Sitting and Lying "Six-Character Formula" breathing Exercise in the early pulmonary rehabilitation of Chronic Obstructive Pulmonary Disease | 🞎 | 🞎 | 🞎 | 🞎 | 🞎 |  |
|  |  | 2.4.3 The application of the Eight-Method Acupoint opening Technique of Linggui in patients with cough and asthma caused by wind-cold and phlegm-dampness | 🞎 | 🞎 | 🞎 | 🞎 | 🞎 |  |
|  |  | 2.4.4 The Application of Gua Sha Therapy in respiratory function Exercise for patients with chronic obstructive pulmonary disease | 🞎 | 🞎 | 🞎 | 🞎 | 🞎 |  |
|  |  | Comments： | | | | | | |
|  | 2.5 Traditional Chinese medicine nursing during the recovery and acute phases of cerebral infarction | 2.5.1 The application of Fire Dragon Cupping in Patients with hemiplegia after stroke | 🞎 | 🞎 | 🞎 | 🞎 | 🞎 |  |
|  |  | 2.5.2 The application of external application of Bingxiao Powder in the prevention of deep vein thrombosis | 🞎 | 🞎 | 🞎 | 🞎 | 🞎 |  |
|  |  | 2.5.3The application of applying nitroyellow powder to the navel in patients with stroke due to excessive heat and constipation | 🞎 | 🞎 | 🞎 | 🞎 | 🞎 |  |
|  |  | 2.5.4The Application of Traditional Chinese Medicine Rewei in Patients with hemiplegia after stroke | 🞎 | 🞎 | 🞎 | 🞎 | 🞎 |  |
|  |  | 2.5.5Clinical Application of acupoint massage along meridians in the rehabilitation of hemiplegic patients after stroke | 🞎 | 🞎 | 🞎 | 🞎 | 🞎 |  |
|  |  | 2.5.6The application of Traditional Chinese medicine hot compress in improving hemiplegia Symptoms in stroke patients | 🞎 | 🞎 | 🞎 | 🞎 | 🞎 |  |
|  |  | 2.5.7The application of moxibustion with moxa sticks in improving urinary and fecal incontinence in stroke patients | 🞎 | 🞎 | 🞎 | 🞎 | 🞎 |  |
|  |  | 2.5.8The application of Fire Dragon Cupping Combined with acupoint bloodletting in improving limb swelling in Stroke patients | 🞎 | 🞎 | 🞎 | 🞎 | 🞎 |  |
|  |  | 2.5.9The Application of auricular therapy in Complications of Stroke patients | 🞎 | 🞎 | 🞎 | 🞎 | 🞎 |  |
|  |  | Comments： | | | | | | |
|  | 2.6 Traditional Chinese Medicine Nursing for anal fistula | 2.6.1 Clinical application of moxibustion combined with auricular point therapy in patients with urinary retention after anal fistula surgery | 🞎 | 🞎 | 🞎 | 🞎 | 🞎 |  |
|  |  | 2.6.2 The clinical application of Lu's moxibustion device in perianal wounds | 🞎 | 🞎 | 🞎 | 🞎 | 🞎 |  |
|  |  | 2.6.3 Clinical application of traditional Chinese medicine mud moxibustion in the treatment of postoperative urinary retention after benign anorectal diseases | 🞎 | 🞎 | 🞎 | 🞎 | 🞎 |  |
|  |  | 2.6.4Clinical application of Bian Shi hot rubbing or hot rubbing in defecation after anal fistula surgery | 🞎 | 🞎 | 🞎 | 🞎 | 🞎 |  |
|  |  | 2.6.5Clinical Application of Traditional Chinese medicine fumigation and washing in perianal diseases | 🞎 | 🞎 | 🞎 | 🞎 | 🞎 |  |
|  |  | 2.6.6Clinical application of apical bloodletting in anal distension after anal fistula surgery | 🞎 | 🞎 | 🞎 | 🞎 | 🞎 |  |
|  |  | Comments： | | | | | | |
|  | 2.7Preventive care in traditional Chinese medicine | 2.7.1 An overview of the theory of preventive medicine and proactive health awareness | 🞎 | 🞎 | 🞎 | 🞎 | 🞎 |  |
|  |  | 2.7.2 Prevention before illness and prevention of disease progression once it occurs | 🞎 | 🞎 | 🞎 | 🞎 | 🞎 |  |
|  |  | Comments： | | | | | | |
|  | 2.8Traditional Chinese medicine home health preservation care   3.1 The clinical syndrome differentiation application of common appropriate techniques in traditional Chinese medicine | 2.8.1Self-care for sub-health with traditional Chinese medicine | 🞎 | 🞎 | 🞎 | 🞎 | 🞎 |  |
|  |  | 2.8.2Self-health care techniques for traditional Chinese medicine at home | 🞎 | 🞎 | 🞎 | 🞎 | 🞎 |  |
|  |  | Comments： | | | | | | |
|  |  |  |  |  |  |  |  |  |
|  |  | 3.1.1The principles and characteristics of syndrome differentiation techniques | 🞎 | 🞎 | 🞎 | 🞎 | 🞎 |  |
|  |  | 3.1.2 The clinical syndrome differentiation application of gua sha technique | 🞎 | 🞎 | 🞎 | 🞎 | 🞎 |  |
|  |  | 3.1.3 Clinical syndrome differentiation application of moxibustion (including Fire Dragon Moxibustion, Umbilical moxibustion, Du Meridian Moxibustion, etc.) | 🞎 | 🞎 | 🞎 | 🞎 | 🞎 |  |
|  |  | 3.1.4 Clinical syndrome differentiation applications of cupping techniques (such as needling cupping, balanced cupping, warming cupping, earthenware cupping, etc.) | 🞎 | 🞎 | 🞎 | 🞎 | 🞎 |  |
|  |  | 3.1.5Clinical syndrome differentiation Application of acupoint application (including summer treatment for winter diseases) | 🞎 | 🞎 | 🞎 | 🞎 | 🞎 |  |
|  |  | 3.1.6The clinical syndrome differentiation application of the hot compress method of traditional Chinese medicine | 🞎 | 🞎 | 🞎 | 🞎 | 🞎 |  |
|  |  | 3.1.7The clinical syndrome differentiation application of the method of rubbing and massaging traditional Chinese medicine with Bian stone | 🞎 | 🞎 | 🞎 | 🞎 | 🞎 |  |
|  |  | 3.1.8The clinical dialectical application of fire ironing | 🞎 | 🞎 | 🞎 | 🞎 | 🞎 |  |
|  |  | 3.1.9The clinical dialectical application of wrist and ankle acupuncture | 🞎 | 🞎 | 🞎 | 🞎 | 🞎 |  |
|  |  | 3.1.10The clinical syndrome differentiation application of auricular point pressing | 🞎 | 🞎 | 🞎 | 🞎 | 🞎 |  |
|  |  | 3.1.11The clinical syndrome differentiation application of bloodletting therapy (such as auricular bloodletting, etc.) | 🞎 | 🞎 | 🞎 | 🞎 | 🞎 |  |
|  |  | 3.1.12Clinical syndrome differentiation application of traditional Chinese medicine transdelivery method (iontophoresis) | 🞎 | 🞎 | 🞎 | 🞎 | 🞎 |  |
|  |  | 3.1.13The clinical dialectical application of pediatric tuina techniques | 🞎 | 🞎 | 🞎 | 🞎 | 🞎 |  |
|  |  | Comments： | | | | | | |
|  |  |  | | | | | | |
|  | 3.2  Clinical care based on syndrome differentiation for common diseases | 3.2.1 Diagnosis and care for common heart and brain diseases | 🞎 | 🞎 | 🞎 | 🞎 | 🞎 |  |
|  |  | 3.2.2 Diagnosis and care for common lung diseases and syndromes | 🞎 | 🞎 | 🞎 | 🞎 | 🞎 |  |
|  |  | 3.2.3 Diagnosis and protection for common diseases of the spleen and stomach | 🞎 | 🞎 | 🞎 | 🞎 | 🞎 |  |
|  |  | 3.2.4 Diagnosis and care for common diseases and syndromes of the liver, gallbladder, kidneys and bladder | 🞎 | 🞎 | 🞎 | 🞎 | 🞎 |  |
|  |  | 3.2.5 Diagnosis and care for common diseases and syndromes of qi, blood, body fluids and body fluids | 🞎 | 🞎 | 🞎 | 🞎 | 🞎 |  |
|  |  | 3.2.6 Diagnosis and protection for common diseases and syndromes of limb meridians | 🞎 | 🞎 | 🞎 | 🞎 | 🞎 |  |
|  |  | 3.2.7 Diagnosis and care for common surgical diseases and syndromes | 🞎 | 🞎 | 🞎 | 🞎 | 🞎 |  |
|  |  | 3.2.8 Diagnosis and care for common gynecological diseases | 🞎 | 🞎 | 🞎 | 🞎 | 🞎 |  |
|  |  | 3.2.9 Diagnosis and care for common diseases and syndromes during pregnancy and after childbirth | 🞎 | 🞎 | 🞎 | 🞎 | 🞎 |  |
|  |  | 3.2.10 Diagnosis and care for common pediatric diseases and syndromes | 🞎 | 🞎 | 🞎 | 🞎 | 🞎 |  |
|  |  | Comments： | | | | | | |
|  |  |  | | | | | | |
| 4.Professional development ability | 4.1 Nursing research capacity | 4.1.1 Writing and defense skills for nursing papers | 🞎 | 🞎 | 🞎 | 🞎 | 🞎 |  |
|  |  | 4.1.2 Cultivation and application of evidence-based Nursing ability | 🞎 | 🞎 | 🞎 | 🞎 | 🞎 |  |
|  |  | 4.1.3 Writing skills for case nursing papers | 🞎 | 🞎 | 🞎 | 🞎 | 🞎 |  |
|  |  | 4.1.4The mining and application of big data in clinical Traditional Chinese medicine nursing | 🞎 | 🞎 | 🞎 | 🞎 | 🞎 |  |
|  |  | 4.1.5Patent application and conversion | 🞎 | 🞎 | 🞎 | 🞎 | 🞎 |  |
|  |  | Comments： | | | | | | |
|  |  |  | | | | | | |
|  |  |  | | | | | | |
|  | 4.2 teaching ability  4.3 innovation ability | 4.2.1 Characteristics and Innovations of Clinical Nursing Teaching in Traditional Chinese Medicine | 🞎 | 🞎 | 🞎 | 🞎 | 🞎 |  |
|  |  | 4.2.2 Strategies for Enhancing Clinical Nursing Teaching Competence | 🞎 | 🞎 | 🞎 | 🞎 | 🞎 |  |
|  |  | 4.2.3Implementation and application of clinical teaching in Traditional Chinese medicine | 🞎 | 🞎 | 🞎 | 🞎 | 🞎 |  |
|  |  |  | | | | | | |
|  |  | 4.3.1The cultivation of innovative thinking ability in Traditional Chinese medicine nursing | 🞎 | 🞎 | 🞎 | 🞎 | 🞎 |  |
|  |  | 4.3.2Apply the new concepts, knowledge and technologies of traditional Chinese medicine nursing to guide nursing practice | 🞎 | 🞎 | 🞎 | 🞎 | 🞎 |  |
|  |  | 4.3.3The formulation and implementation of new standards and norms for traditional Chinese medicine nursing | 🞎 | 🞎 | 🞎 | 🞎 | 🞎 |  |
|  |  | 4.3.4Inherit and innovate traditional Chinese medicine nursing techniques and the innovation of tools | 🞎 | 🞎 | 🞎 | 🞎 | 🞎 |  |
|  |  | 4.3.5The Application of Smart Information-based Nursing in Clinical Nursing of Traditional Chinese Medicine | 🞎 | 🞎 | 🞎 | 🞎 | 🞎 |  |
|  |  | 4.3.6 The improvement and innovation of the scope of application and service methods of traditional Chinese medicine nursing | 🞎 | 🞎 | 🞎 | 🞎 | 🞎 |  |
|  |  | Comments： | | | | | | |
|  |  |  | | | | | | |
|  | 4.4 critical thinking | 4.4.1The Cultivation of critical thinking in clinical nursing of Traditional Chinese Medicine | 🞎 | 🞎 | 🞎 | 🞎 | 🞎 |  |
|  |  | 4.4.2The Application of Critical Thinking in Nursing Teaching and Research | 🞎 | 🞎 | 🞎 | 🞎 | 🞎 |  |
|  |  | 4.4.3The Application of Critical Thinking in Clinical Nursing Decision-making | 🞎 | 🞎 | 🞎 | 🞎 | 🞎 |  |
|  |  | Comments： | | | | | | |
|  |  |  | | | | | | |
|  | 4.5autonomous learning ability | 4.5.1Active learning: New concepts and technologies in Traditional Chinese medicine nursing | 🞎 | 🞎 | 🞎 | 🞎 | 🞎 |  |
|  |  | 4.5.2The implementation of academic exchanges and professional learning | 🞎 | 🞎 | 🞎 | 🞎 | 🞎 |  |
|  |  | 4.5.3 Keep a close eye on the cutting-edge developments in the discipline | 🞎 | 🞎 | 🞎 | 🞎 | 🞎 |  |
| 5.Nursing management ability | 5.1Quality management of Traditional Chinese medicine nursing | 5.1.1 Qualification access and hierarchical management of traditional Chinese medicine nursing techniques | 🞎 | 🞎 | 🞎 | 🞎 | 🞎 |  |
|  |  | 5.1.2 Evaluation indicators of clinical nursing quality in traditional Chinese medicine | 🞎 | 🞎 | 🞎 | 🞎 | 🞎 |  |
|  |  | 5.1.3Selection of evaluation indicators for the therapeutic effects of traditional Chinese medicine techniques | 🞎 | 🞎 | 🞎 | 🞎 | 🞎 |  |
|  |  | 5.1.4Establishment and supervision of safety awareness in Traditional Chinese medicine nursing | 🞎 | 🞎 | 🞎 | 🞎 | 🞎 |  |
|  |  | 5.1.5The Application of Common management Tools in the Continuous Improvement of Traditional Chinese Medicine Nursing Quality | 🞎 | 🞎 | 🞎 | 🞎 | 🞎 |  |
|  |  | 5.1.6Occupational protection in traditional Chinese medicine nursing | 🞎 | 🞎 | 🞎 | 🞎 | 🞎 |  |
|  |  | 5.1.7Quality evaluation of Satisfaction with Traditional Chinese Medicine Nursing services |  |  |  |  |  |  |
|  |  | Comments： | | | | | | |
|  | 5.2 Organizational and leadership skills | 5.2.1 The cultivation of leadership skills in traditional Chinese medicine nursing | 🞎 | 🞎 | 🞎 | 🞎 | 🞎 |  |
|  |  | 5.2.2 The organization and operation of the team specializing in traditional Chinese medicine | 🞎 | 🞎 | 🞎 | 🞎 | 🞎 |  |
|  |  | 5.2.3The cultivation and improvement of specialized nursing talents in traditional Chinese medicine | 🞎 | 🞎 | 🞎 | 🞎 | 🞎 |  |
|  |  | Comments： | | | | | | |
|  | 5.3 Communication, coordination and cooperation skills | 5.3.1The basic principles of communication and coordination | 🞎 | 🞎 | 🞎 | 🞎 | 🞎 |  |
|  |  | 5.3.2 The ability to communicate efficiently with patients and their families and handle nurse-patient conflicts has been enhanced | 🞎 | 🞎 | 🞎 | 🞎 | 🞎 |  |
|  |  | 5.3.3 Medical care and multi-disciplinary professional collaboration capabilities | 🞎 | 🞎 | 🞎 | 🞎 | 🞎 |  |
|  |  | 5.3.4Communication Case sharing | 🞎 | 🞎 | 🞎 | 🞎 | 🞎 |  |
|  |  | Comments： | | | | | | |
| 6.Ethical and moral literacy | 6.1 humanistic care | 6.1.1 Caring as the Foundation: Nurse-patient communication and Humanistic care | 🞎 | 🞎 | 🞎 | 🞎 | 🞎 |  |
|  |  | 6.1.2 Benevolence and skill: Cultivate virtue and style with the culture of traditional Chinese medicine | 🞎 | 🞎 | 🞎 | 🞎 | 🞎 |  |
|  |  | Comments： | | | | | | |
|  |  |  | | | | | | |
|  | 6.2  moral principles | 6.2.1The roots and connotations of ancient Chinese medical ethical thought | 🞎 | 🞎 | 🞎 | 🞎 | 🞎 |  |
|  |  | 6.2.2 Guarding Dignity: Patient Respect and Privacy Protection in Medical Care | 🞎 | 🞎 | 🞎 | 🞎 | 🞎 |  |
|  |  | 6.2.3Informed consent in nursing research and operations | 🞎 | 🞎 | 🞎 | 🞎 | 🞎 |  |
|  |  | 6.2.4The medical ethics and conduct of the medical practitioners in Xin 'an | 🞎 | 🞎 | 🞎 | 🞎 | 🞎 |  |
|  |  | Comments： | | | | | | |
|  |  |  |  |  |  |  |  |  |
|  | 6.3 Medical regulations | 6.3.1 Medical and nursing related laws and regulations | 🞎 | 🞎 | 🞎 | 🞎 | 🞎 |  |
|  |  | 6.3.2 Nursing-related rules and regulations | 🞎 | 🞎 | 🞎 | 🞎 | 🞎 |  |
|  |  | 6.3.3 Relevant norms and standards for traditional Chinese medicine nursing | 🞎 | 🞎 | 🞎 | 🞎 | 🞎 |  |
|  |  | Comments： |  |  |  |  |  |  |
